# Supplementary material for: Do exhausted primary school students cheat more? A randomized field experiment
Source: PLoS One. 2021 Dec 1;16(12):e0260141. doi: 10.1371/journal.pone.0260141 (PMC8635394; doi:10.1371/journal.pone.0260141)
Supplement: S1 Table — (DOCX) [file pone.0260141.s001.docx]

**S1 Table: Mean differences in students’ baseline grades between those who answered/did not answer the online survey**

|  | (1) | (2) | (3) | (4) | (5) | (6) | (7) |
| --- | --- | --- | --- | --- | --- | --- | --- |
|  | Mathematics | Literature | Grammar | History | Foreign language | Behavior | Diligence |
| Mean difference: answered/did not answer | 0.449** | 0.358** | 0.393** | 0.405** | 0.399** | 0.242** | 0.360** |
|  | (0.045) | (0.040) | (0.046) | (0.052) | (0.044) | (0.042) | (0.040) |
| Constant (did not answer) | 3.337** | 3.700** | 3.536** | 3.511** | 3.655** | 4.127** | 3.830** |
|  | (0.017) | (0.015) | (0.017) | (0.020) | (0.017) | (0.016) | (0.015) |
| Observations | 2,872 | 2,893 | 2,893 | 1,893 | 2,552 | 2,885 | 2,885 |
| R-squared | 0.227 | 0.316 | 0.294 | 0.300 | 0.284 | 0.251 | 0.288 |

Standard errors in parentheses, ** p<0.01, * p<0.05, + p<0.1

Each model contains classroom fixed effects. Standard errors are clustered at the school level

Grades are teacher-reported and refer to end-of-term grades in the academic year 2018/19 (the last academic year before the online survey)
