# Supplementary material for: Do exhausted primary school students cheat more? A randomized field experiment
Source: PLoS One. 2021 Dec 1;16(12):e0260141. doi: 10.1371/journal.pone.0260141 (PMC8635394; doi:10.1371/journal.pone.0260141)
Supplement: S2 Table — (DOCX) [file pone.0260141.s002.docx]

**S2 Table: Differences between students in schools that are/are not in our experiment based on 6^th^-grade students’ data in a nationwide administrative dataset**

|  | (1) | (2) | (3) |
| --- | --- | --- | --- |
|  | Mean in schools NOT in the sample  N of schools = 2,191 | Mean in schools in the sample  N of schools = 28 | Mean difference^(a)^ |
| Test score in mathematics | 0.004 | -0.242 | -0.246*** |
| Test score in reading | 0.004 | -0.25 | -0.254** |
| Mother has university degree | 0.311 | 0.207 | -0.104** |
| Mother has a job | 0.778 | 0.76 | -0.017 |
| Father has university degree | 0.236 | 0.154 | -0.082** |
| Father has a job | 0.873 | 0.861 | -0.012 |
| Number of books at home | 0.003 | -0.175 | -0.178* |

^(a)^ Standard errors are clustered at the school level

Robust standard errors in parentheses, *** p<0.001, ** p<0.01, * p<0.05

The source of data is the NABC database, which contains data on 6th-grade students based on a yearly assessment of the corresponding 6th-grade cohort. We used the 6th cohort in 2017 (the latest publicly available NABC dataset) in our calculations. We were able to identify participating schools but not participating students in the NABC data. Statistics in the table may not correspond to the 6th grade students in our sample.

N of students is the NABC database = 91,599
