# Supplementary material for: Do exhausted primary school students cheat more? A randomized field experiment
Source: PLoS One. 2021 Dec 1;16(12):e0260141. doi: 10.1371/journal.pone.0260141 (PMC8635394; doi:10.1371/journal.pone.0260141)
Supplement: S3 Table — (DOCX) [file pone.0260141.s003.docx]

**S3 Table: Balance in the sample: Mean of baseline variables in the control group and treated group relative to the control group**

|  | (1) | (2) | (3) | (4) |
| --- | --- | --- | --- | --- |
|  | Control group | Treated group relative to control group | | N |
|  |  | Simple difference | controlling for classroom fixed effects^(a)^ |  |
| Girl | 0.556 | -0.084** | -0.078** | 1,143 |
| Age | 12.84 | -0.023 | -0.004 | 1,143 |
| N of books | 0.038 | -0.054 | -0.050 | 1,106 |
| GPA (January 2020) | 3.746 | -0.014 | -0.008 | 1,127 |
| Disruptive school behavior (February 2020) | 1.315 | 0.024 | 0.012 | 1,025 |
| Math test score (April 2020) | 0.69 | -0.022 | -0.021 | 983 |
| Delay of gratification (April 2020) | 0.835 | -0.031 | -0.035 | 983 |
| Altruism (April 2020) | 0.848 | 0.007 | 0.010 | 983 |

The difference between the means of the treated and control group is calculated with regression analysis, where the corresponding variable in the rows of the table (dependent variable) was regressed on a dummy variable marking the treatment status (=1 if students are in the treatment group). Standard errors are clustered at the school level.

^(a)^ The regressions deployed classroom fixed-effects.

Robust standard errors in parentheses; ** p<0.01, * p<0.05, + p<0.1
