# Supplementary material for: Do exhausted primary school students cheat more? A randomized field experiment
Source: PLoS One. 2021 Dec 1;16(12):e0260141. doi: 10.1371/journal.pone.0260141 (PMC8635394; doi:10.1371/journal.pone.0260141)
Supplement: S4 Table — (DOCX) [file pone.0260141.s004.docx]

**S4 Table: Results of regression analysis with conditional logit model, logit coefficients**

|  | (1) | (2) | (3) | (4) | (5) | (6) |
| --- | --- | --- | --- | --- | --- | --- |
|  | misreported dice roll | | opted for a more valuable object | | opted for a less valuable object | |
|  | Control: preregistered | Control:  full | Control: preregistered | Control:  full | Control: preregistered | Control:  full |
| Treated | 0.451+ | 0.457+ | 0.648** | 0.696** | 0.051 | -0.048 |
|  | (0.244) | (0.248) | (0.239) | (0.241) | (0.459) | (0.479) |
| Observations | 1,143 | 1,143 | 1,096 | 1,096 | 1,046 | 1,046 |

All models contain constant, classroom-fixed effects and the preregistered control variables: gender and age. Standard errors are clustered at the school level.

The list of baseline control variables in *full* specifications is as follows: gender, age, N of books, GPA, teacher-reported disruptive school behavior, math test, delay of gratification (DG), baseline altruism. Missing values in baseline control variables have been replaced with 0, and separate dummy variables control for missing status. Descriptive statistics and the coding of baseline control variables are shown in Table 1
