# Supplementary material for: Do exhausted primary school students cheat more? A randomized field experiment
Source: PLoS One. 2021 Dec 1;16(12):e0260141. doi: 10.1371/journal.pone.0260141 (PMC8635394; doi:10.1371/journal.pone.0260141)
Supplement: S1 Appendix — (DOCX) [file pone.0260141.s005.docx]

# **S1 Appendix: sample questions used in the math test**

*The two sample questions are from the test for 4^th^ graders*

**John tries to get to the bank shown in the picture by car.**


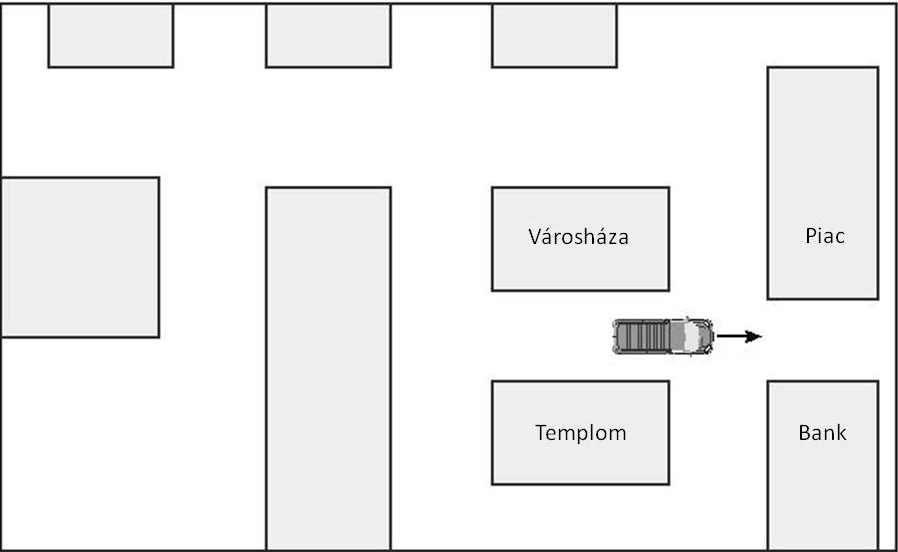


Bank

Market

Church

Town hall

**
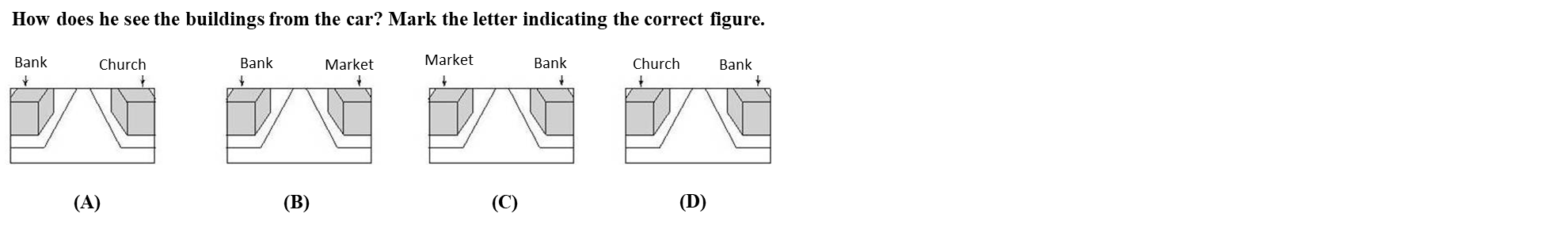
**

**CNN filmed the men’s 1,500 meters race in Oskeiland from four camera angles. The following figure shows the positions of the four runners labeled 1, 2, 3, and 4, and the locations of cameras A, B, C, and D.**

Direction of run


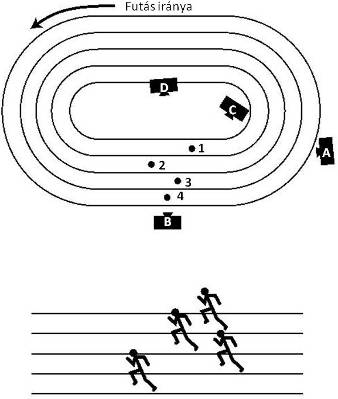


**Which camera recorded the following picture of the position of the runners? Mark the letter indicating the correct answer.**


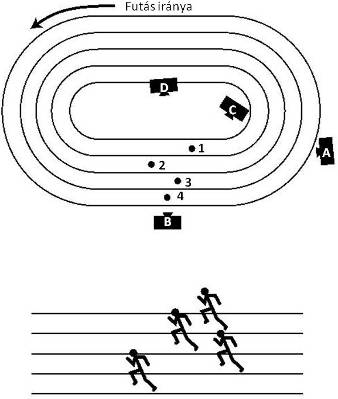


**(A) (B) (C) (D)**
