# Supplementary material for: Do exhausted primary school students cheat more? A randomized field experiment
Source: PLoS One. 2021 Dec 1;16(12):e0260141. doi: 10.1371/journal.pone.0260141 (PMC8635394; doi:10.1371/journal.pone.0260141)
Supplement: Attachment — Submitted filename: Response to Reviewers.docx [file pone.0260141.s007.docx]

Journal Requirements:

Please review your reference list to ensure that it is complete and correct. If you have cited papers that have been retracted, please include the rationale for doing so in the manuscript text, or remove these references and replace them with relevant current references. Any changes to the reference list should be mentioned in the rebuttal letter that accompanies your revised manuscript. If you need to cite a retracted article, indicate the article’s retracted status in the References list and also include a citation and full reference for the retraction notice.

.

**We do not cite Ariely’s retracted paper (Signing at the beginning makes ethics salient and decreases dishonest self-reports in comparison to signing at the end), published in PNAS and retracted in September 2021. The retracted paper concerns cheating from the perspective of self-concept maintenance theory and not from ego-depletion theory. Therefore, we have not cited this research in any of the earlier versions of this manuscript.**

Reviewer’s comment:

The authors paid attention to my previous comments and did a good job in revising the paper.

Please notice that on line 311 you refer to Table 1, whereas the correct reference is now Table 2.

**Thank you very much for your careful reading. We have corrected our mistake in line 311.**
